# Factors associated with interferon-gamma release assay positivity in patients with psoriasis: a cross-sectional study

**DOI:** 10.1186/s12879-026-14090-z

**Published:** 2026-07-29

**Authors:** Hewei Song, Yao Zhang, Qingzhu Zhang, Jin Zhang, Weijie Gu, Pei Yang, Yang Xiao, Fang Liu, Min Li, Wenjing Ding, Kexu Song, Lina Qiao, Yanhua Li, Yueyun Ma

**Affiliations:** 1 Department of Clinical Laboratory, Air Force Medical Center, Air Force Medical University, Beijing, 100142 China; 2 Department of Blood Quality Control, Yantai Central Blood Station, Yantai, Shandong Province 264003 China; 3 Department of Dermatology, Air Force Medical Center, Air Force Medical University, Beijing, 100142 China

**Keywords:** Psoriasis, Interferon-gamma release assay, Tuberculosis, Latent tuberculosis infection, Predictive model, Risk factors

## Abstract

**Background:**

The interferon-gamma release assay (IGRA) is recommended for latent tuberculosis infection (LTBI) screening prior to biologic therapy in psoriasis. Yet factors influencing IGRA positivity in this population remain insufficiently characterized.

**Objectives:**

This study aimed to identify clinical factors associated with IGRA positivity among patients with psoriasis and to develop an internally validated model to summarize these associations in routine dermatological care.

**Methods:**

We retrospectively analysed 1,126 patients with psoriasis without a documented history of prior tuberculosis (285 IGRA-positive and 841 IGRA-negative) at a single dermatology centre. Variables associated with IGRA positivity in univariable analysis were entered into a multivariable logistic regression model, which was internally validated using bootstrap resampling.

**Results:**

The overall IGRA positivity rate was 25.3%. IGRA positivity increased markedly with age, from 8.2% in patients aged ≤ 30 years to 45.6% in those aged > 60 years, with an optimal ROC-derived cutoff of 49 years (sensitivity, 0.561; specificity, 0.762). In multivariable analysis, older age (OR 1.048, 95% CI 1.037–1.059, *p* < 0.001), pulmonary nodules (OR 1.608, 95% CI 1.094–2.365, *p* = 0.016), and higher eosinophil percentage (OR 1.109, 95% CI 1.033–1.190, *p* = 0.004) were independently associated with increased odds of IGRA positivity, whereas prior biologic exposure was associated with reduced odds (OR 0.705, 95% CI 0.512–0.969, *p* = 0.031). The eight-variable multivariable model demonstrated acceptable discrimination (AUC 0.726; optimism-corrected AUC 0.717, 95% CI 0.690–0.763) and good calibration (Brier score 0.167). Decision curve analysis suggested net clinical benefit across a threshold range of approximately 5%–50%.

**Conclusions:**

Older age, pulmonary nodules, and higher eosinophil percentage were independently associated with IGRA positivity, while prior biologic exposure showed an inverse association likely related to selection bias. The model showed acceptable internal performance and may provide supplementary context, but should not replace standard pre-biologic tuberculosis assessment.

**Supplementary Information:**

The online version contains supplementary material available at 10.1186/s12879-026-14090-z.

## Significance

**What is already known about this topic?**

1. Screening for latent tuberculosis infection (LTBI) is recommended prior to biologic therapy in patients with psoriasis because certain agents, particularly TNF-α inhibitors, may increase the risk of tuberculosis reactivation.

2. Interferon-gamma release assays (IGRAs) are widely used for LTBI detection and are preferred over the tuberculin skin test in populations with high Bacillus Calmette–Guérin vaccination coverage due to their higher specificity.

3. Reported IGRA positivity rates in psoriasis vary substantially across studies, reflecting differences in tuberculosis endemicity and patient characteristics.

4. However, the independent clinical factors associated with IGRA positivity in psoriasis remain incompletely defined, and few internally validated models have been reported for estimating the probability of IGRA positivity during tuberculosis assessment in routine dermatological care.

**What does this study add?**

1. This study identifies older age, pulmonary nodules, and higher blood eosinophil percentage as independent clinical factors associated with IGRA positivity in patients with psoriasis without prior tuberculosis history.

2. It develops an internally validated multivariable model with acceptable discrimination and calibration, providing a quantitative framework for estimating the probability of IGRA positivity during tuberculosis assessment in routine dermatological care.

3. Prior biologic exposure was associated with lower odds of IGRA positivity, a finding most likely explained by clinical management pathways and selection bias rather than a true protective effect.

4. Differences in IGRA positivity across psoriasis subtypes were observed, suggesting that disease phenotype may contribute to heterogeneity in LTBI screening outcomes.

**Supplementary Information:**

The online version contains supplementary material available at 10.1186/s12879-026-14090-z.

## Introduction

Psoriasis is a chronic immune-mediated inflammatory skin disease affecting approximately 2–3% of the global population [1, 2]. The advent of biologic therapies—including TNF-α inhibitors, IL-17 inhibitors, and IL-23 inhibitors—has dramatically improved disease management [3, 4]. However, because some biologic agents, particularly TNF-α inhibitors, may increase the risk of tuberculosis reactivation, tuberculosis infection assessment has become an important component of clinical management for patients with psoriasis receiving or being considered for biologic therapy [5–7].

The interferon-gamma release assay (IGRA), which measures T-cell responses to Mycobacterium tuberculosis-specific antigens such as ESAT-6 and CFP-10, is widely recommended for latent tuberculosis infection (LTBI) screening [8]. Unlike the tuberculin skin test (TST), IGRA is not affected by prior Bacillus Calmette-Guérin (BCG) vaccination and has greater specificity in BCG-vaccinated populations, making it particularly useful in regions with high BCG coverage such as East Asia [9, 10]. Reported IGRA positivity rates in psoriasis cohorts vary substantially across studies and geographic regions, likely reflecting differences in tuberculosis endemicity, BCG vaccination practices, treatment exposure, and patient characteristics [11, 12].

Despite its established utility, IGRA positivity should not be interpreted as an isolated laboratory finding or as a direct substitute for clinical risk assessment. IGRA results may be influenced by host characteristics beyond underlying LTBI status [13]. Prior studies in different populations have shown associations of IGRA outcomes with age, metabolic comorbidities, renal dysfunction, and selected hematological parameters [14–16]. In the specific context of psoriasis—a disease characterized by systemic inflammatory dysregulation with prominent Th1/Th17 pathways—the immunological milieu may further influence IGRA performance and positivity rates [17]. Alterations in eosinophil levels may reflect broader immune dysregulation and could potentially modulate host responses to tuberculosis antigens, although this possibility has not been systematically examined in patients with psoriasis.

This issue is particularly relevant in regions with an intermediate or high tuberculosis burden. In such settings, a relatively high IGRA positivity rate may reflect the underlying epidemiological background and the real-world characteristics of dermatology patients undergoing tuberculosis infection assessment. Therefore, beyond simply reporting the prevalence of IGRA positivity, it is important to clarify which clinical features are associated with IGRA-positive results and whether these factors can be integrated into a practical risk assessment framework to support the interpretation of IGRA findings and patient management.

Comprehensive analysis of the independent clinical determinants of IGRA positivity in patients with psoriasis remains limited. Understanding these factors is clinically significant: overestimation of tuberculosis infection risk may lead to unnecessary prophylactic therapy with associated hepatotoxicity risks, whereas underestimation may contribute to tuberculosis reactivation during biologic therapy [18]. Furthermore, to our knowledge, few studies have developed and internally validated a practical model to quantify individual IGRA positivity risk in real-world psoriasis populations, including patients with varied treatment exposure. Therefore, this retrospective cross-sectional study aimed to systematically investigate demographic, clinical, and laboratory factors associated with IGRA positivity in a large cohort of patients with psoriasis, and to develop and internally validate a model that summarizes these associations. The model was intended to provide supplementary context for interpreting IGRA results in the setting of biologic therapy evaluation, rather than to replace routine IGRA testing, chest imaging, tuberculosis history assessment, or guideline-based clinical decision-making.

## Methods

### Study design and participants

We conducted a retrospective cross-sectional study at the Air Force Medical Center in Beijing, China. Patients with a confirmed diagnosis of psoriasis who underwent IGRA testing between January 2023 and August 2025 were enrolled. Eligible patients included those who received IGRA testing as part of routine pre-biologic therapy evaluation or clinical follow-up. Patients with indeterminate IGRA results (*n* = 2) and those with a documented history of prior tuberculosis (*n* = 10) were excluded, yielding a final analytic cohort of 1,126 participants. Patients with a history of prior tuberculosis were excluded because IGRA results in this subgroup predominantly reflect known previous infection rather than the screening scenario in which the model is intended to be applied. The study protocol was approved by the Institutional Review Board of the Air Force Medical Center (approval No. 2023-41-YJ103, with annual renewal in January 2026), and the requirement for written informed consent was waived owing to the retrospective nature of the study. All procedures were conducted in accordance with the Declaration of Helsinki.

### IGRA testing

IGRA was performed using the Mycobacterium tuberculosis-Specific T Cell Detection Kit (enzyme-linked immunosorbent assay [ELISA]; Beijing Wantai Biological Pharmacy Enterprise Co., Ltd., Beijing, China; NMPA approval No. 20163400981) according to the manufacturer’s instructions. Briefly, heparinized whole-blood samples were incubated at 37 °C for 16–24 h with M. tuberculosis-specific peptide antigens (ESAT-6 and CFP-10) in the antigen tube (T tube), with no antigen in the negative-control tube (N tube), and with a mitogen in the positive-control tube (M tube). Following incubation, plasma supernatants were collected, and IFN-γ concentrations were measured using ELISA. Results were interpreted according to the difference in IFN-γ concentrations between the antigen-stimulated tube and the negative-control tube. A T–N value > 14 pg/mL was defined as positive, whereas a value ≤ 14 pg/mL was defined as negative. The mitogen tube served as an internal positive control. Results were considered indeterminate when quality-control criteria were not met, including a high background response in the negative control or an insufficient response in the mitogen control. Indeterminate results were excluded from further analysis.

### Data collection

The following variables were extracted from medical records: (1) demographics: age, sex, body mass index (BMI), and BMI category; (2) psoriasis-related factors: disease duration in years and categorical groups (≤ 5, 6–10, 11–20, and > 20 years), disease duration ≥ 10 years, biologic therapy type, prior biologic exposure, and psoriasis subtype; (3) laboratory parameters: complete blood count, including white blood cell count (WBC), red blood cell count (RBC), hemoglobin, hematocrit, mean corpuscular volume (MCV), mean corpuscular hemoglobin (MCH), mean corpuscular hemoglobin concentration (MCHC), red cell distribution width (RDW-CV), platelet count, plateletcrit (PCT), mean platelet volume (MPV), platelet distribution width (PDW), and differential counts, including neutrophil, lymphocyte, monocyte, eosinophil, and basophil percentages and absolute counts; and (4) comorbidities: hypertension, diabetes mellitus, fatty liver disease, hyperlipidemia, pulmonary nodules, and liver dysfunction. Prior biologic exposure was defined as documented use of any biologic agent before the index IGRA test. “Any comorbidity” was defined as the presence of at least one of these recorded variables. Psoriasis subtypes were classified according to the primary diagnosis recorded in the medical records, with each patient assigned to one mutually exclusive subtype category. For patients with overlapping psoriasis-related phenotypes, the dominant clinical diagnosis recorded by dermatologists was used for classification. Pulmonary nodules were defined according to chest imaging reports recorded in the electronic medical records. In this study, this variable referred specifically to documented pulmonary nodules, and no other radiographic entities were included under this term.

### Statistical analysis

Continuous variables are expressed as mean ± SD or median (IQR). Categorical variables are reported as frequencies and percentages. Between-group comparisons were performed using the Mann–Whitney U test for continuous variables and the chi-square or Fisher’s exact test for categorical variables. Variables with *p* < 0.05 in univariable logistic regression were considered for entry into the multivariable logistic regression model. The monocyte-to-lymphocyte ratio (MLR) and eosinophil-to-lymphocyte ratio (ELR) were excluded because of collinearity with eosinophil percentage. The final eight-variable model included age, disease duration ≥ 10 years, hypertension, diabetes mellitus, pulmonary nodules, prior biologic exposure, platelet count, and eosinophil percentage; all eight variables were retained when model performance was evaluated. Results are reported as adjusted ORs with 95% CIs. Spearman correlation was used to assess multicollinearity before model construction. Model discrimination was assessed using the AUC, calibration using a calibration curve and Brier score, and internal validity using 1,000-resample bootstrapping. Clinical utility was evaluated using decision curve analysis. The corresponding clinical model excluded the two laboratory variables and included age, disease duration ≥ 10 years, hypertension, diabetes mellitus, pulmonary nodules, and prior biologic exposure. In decision curve analysis, the “treat-all” strategy assumes that all patients are classified as high risk and undergo further tuberculosis-related clinical evaluation, whereas the “treat-none” strategy assumes that no patients are classified as high risk. In this context, “treat” refers to further clinical evaluation rather than anti-tuberculosis treatment. Missing data were handled using multiple imputation by chained equations under the assumption that data were missing at random. All candidate predictors and the outcome were included in the imputation model to minimize potential bias. Multiple imputed datasets were generated, and results were pooled according to Rubin’s rules. All analyses were performed in Python (version 3.11). A two-tailed *p* < 0.05 was considered statistically significant.

## Results

### Patient characteristics, overall IGRA positivity, and age-related trends

A total of 1,138 patients underwent IGRA screening. After excluding 2 patients with indeterminate results and 10 patients with a documented history of prior tuberculosis, 1,126 patients were included in the final analysis, comprising 285 (25.3%) IGRA-positive and 841 (74.7%) IGRA-negative individuals (Table 1). Compared with the IGRA-negative group, IGRA-positive patients were older, had a longer disease duration, and more frequently had pulmonary nodules, hypertension, diabetes mellitus, hyperlipidemia, and at least one comorbidity, whereas prior biologic exposure was less frequent. Psoriasis subtype distribution also differed significantly between the groups, with erythrodermic psoriasis being more common among IGRA-positive patients.

Among laboratory parameters, eosinophil percentage and absolute eosinophil count were both higher in the IGRA-positive group [2.20 (1.40–3.90)% and 0.15 (0.09–0.26) ×10⁹/L, respectively] than in the IGRA-negative group [1.90 (1.20–3.20)% and 0.13 (0.08–0.22) ×10⁹/L, respectively; *p* = 0.004 and *p* = 0.009]. Lymphocyte and platelet counts were lower, whereas MLR and ELR were higher, in IGRA-positive patients; all six between-group differences were statistically significant. WBC count, neutrophil count, NLR, and PLR did not differ significantly between the groups.

**Fig. 1:**
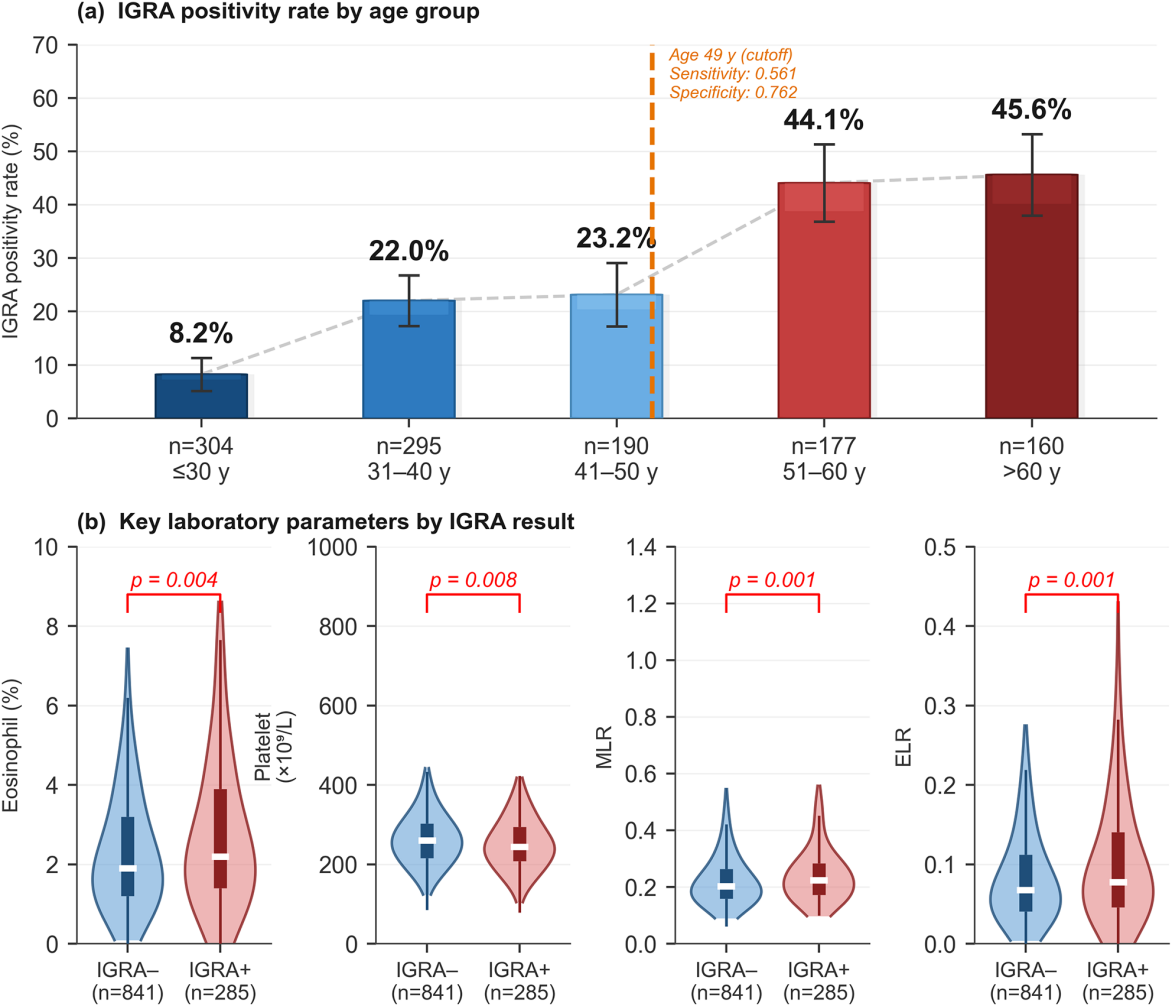
IGRA positivity rate and key laboratory parameters by IGRA result. (**a**) IGRA positivity across different age groups. (**b**) Comparison of eosinophil percentage, platelet count, MLR, and ELR between the IGRA-positive and IGRA-negative groups

IGRA positivity increased markedly with age, rising from 8.2% in patients aged ≤ 30 years to 45.6% in those aged > 60 years (Fig. 1a). ROC analysis identified 49 years as the optimal age cutoff, with a sensitivity of 0.561 and a specificity of 0.762. Differences in eosinophil percentage, platelet count, MLR, and ELR are illustrated in Fig. 1b.

**Table 1:** Baseline characteristics of psoriasis patients stratified by IGRA result

Variable	Total (*n*=1126)	IGRA- (*n*=841)	IGRA+ (*n*=285)	*p* value
***Demographics***				
Age, years	39.0 (29.0–53.0)	36.0 (27.0–48.0)	51.0 (38.0–61.0)	**<0.001**
Male, n (%)	782 (69.4)	571 (67.9)	211 (74.0)	0.061
BMI (kg/m²)	26.07 (23.53–28.20)	26.01 (23.42–28.17)	26.23 (23.66–28.23)	0.551
***Psoriasis characteristics***				
Disease duration, years	10.0 (6.0–15.0)	10.0 (6.0–13.0)	10.0 (8.0–20.0)	**<0.001**
Disease duration ≥10 years, n (%)	750 (66.6)	542 (64.4)	208 (73.0)	**0.010**
Prior biologic exposure, n (%)	828 (73.5)	640 (76.1)	188 (66.0)	**0.001**
Psoriasis subtype, n (%)				**0.034**
Psoriasis vulgaris	1079 (95.8)	808 (96.1)	271 (95.1)	
Erythrodermic psoriasis	20 (1.8)	10 (1.2)	10 (3.5)	
Pustular psoriasis	19 (1.7)	17 (2.0)	2 (0.7)	
Psoriatic arthritis	8 (0.7)	6 (0.7)	2 (0.7)	
***Comorbidities***				
Hypertension, n (%)	128 (11.4)	79 (9.4)	49 (17.2)	**<0.001**
Diabetes mellitus, n (%)	77 (6.8)	45 (5.4)	32 (11.2)	**0.001**
Fatty liver disease, n (%)	150 (13.3)	105 (12.5)	45 (15.8)	0.188
Hyperlipidemia, n (%)	66 (5.9)	42 (5.0)	24 (8.4)	**0.047**
Pulmonary nodules, n (%)	169 (15.0)	101 (12.0)	68 (23.9)	**<0.001**
Liver dysfunction, n (%)	41 (3.6)	32 (3.8)	9 (3.2)	0.748
Any comorbidity, n (%)	336 (29.8)	230 (27.3)	106 (37.2)	**0.002**
***Laboratory parameters***				
WBC (×10⁹/L)	6.84 (5.67–8.17)	6.79 (5.63–8.21)	6.93 (5.71–8.07)	0.906
Neutrophil count (×10⁹/L)	4.14 (3.26–5.22)	4.10 (3.23–5.26)	4.25 (3.34–5.17)	0.614
Lymphocyte count (×10⁹/L)	1.97 (1.56–2.41)	2.00 (1.60–2.41)	1.86 (1.47–2.39)	**0.047**
Eosinophil count (×10⁹/L)	0.14 (0.08–0.23)	0.13 (0.08–0.22)	0.15 (0.09–0.26)	**0.009**
Eosinophil (%)	2.00 (1.30–3.30)	1.90 (1.20–3.20)	2.20 (1.40–3.90)	**0.004**
Platelet (×10⁹/L)	255.0 (215.0–301.0)	260.0 (216.0–303.0)	244.0 (208.0–294.0)	**0.008**
NLR	2.11 (1.54–2.84)	2.08 (1.52–2.78)	2.17 (1.65–2.96)	0.058
PLR	130.2 (101.3–164.4)	130.1 (101.9–163.2)	131.7 (98.5–168.5)	0.894
MLR	0.21 (0.16–0.27)	0.20 (0.16–0.26)	0.22 (0.17–0.28)	**0.001**
ELR	0.07 (0.04–0.12)	0.07 (0.04–0.11)	0.08 (0.05–0.14)	**0.001**

Data presented as median (IQR) or n (%). Patients with indeterminate IGRA results (n = 2) and prior TB history (n = 10) were excluded; final analytic cohort n = 1126. p values: Mann–Whitney U test (continuous); chi-square or Fisher’s exact test (categorical). The P value on the ‘Psoriasis subtype’ row represents the overall between-group chi-square test across all four subtypes. Bold P values: *P* < 0.05. BMI, body mass index; ELR, eosinophil-to-lymphocyte ratio; MLR, monocyte-to-lymphocyte ratio; NLR, neutrophil-to-lymphocyte ratio; PLR, platelet-to-lymphocyte ratio; TB, tuberculosis; WBC, white blood cell count

### Factors associated with IGRA positivity

**Fig. 2:**
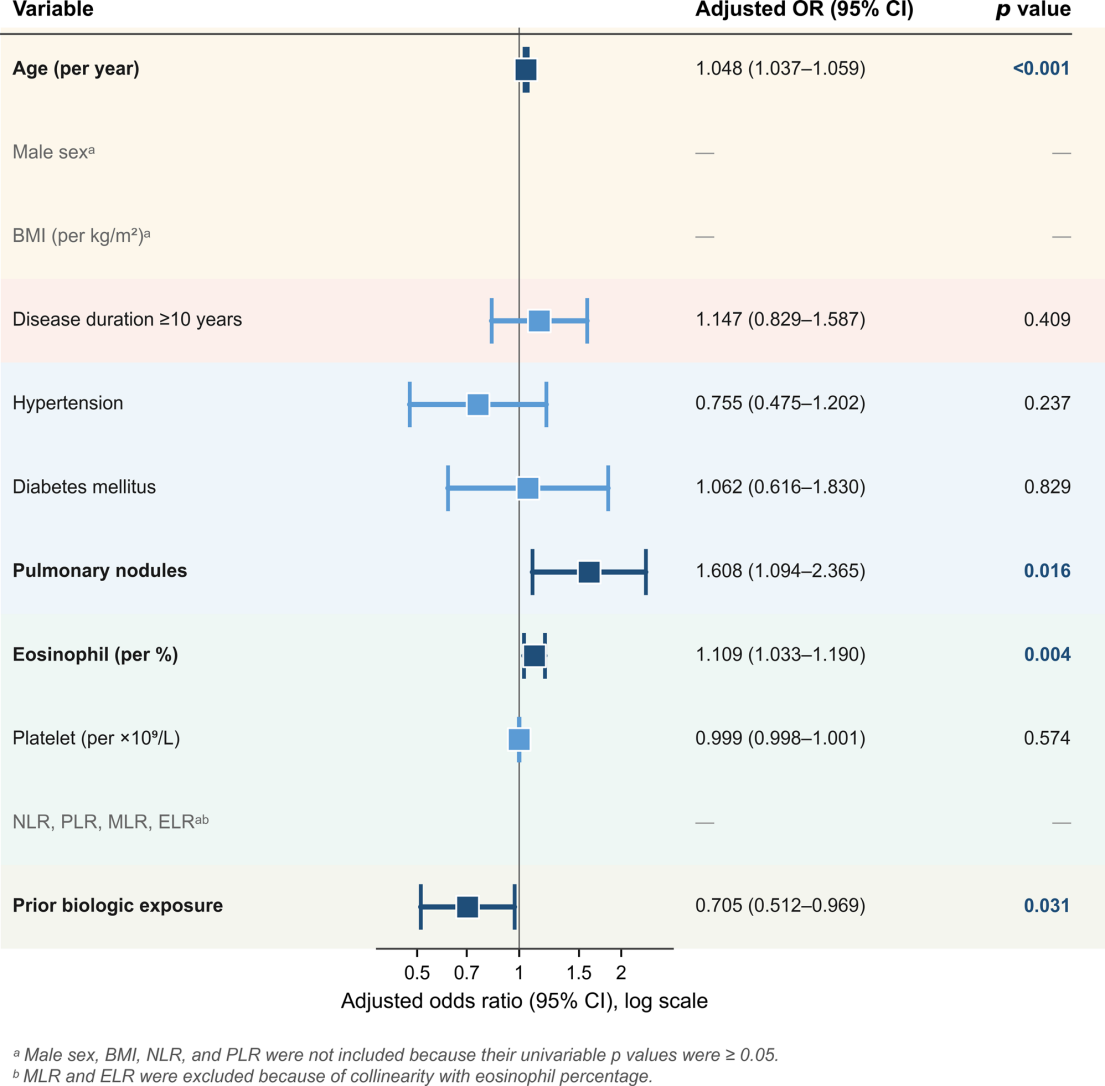
Forest plot of multivariable logistic regression. Forest plot showing adjusted odds ratios and 95% confidence intervals for variables included in the multivariable model. OR, odds ratio; CI, confidence interval; IGRA, interferon-gamma release assay

Several variables were associated with IGRA positivity in univariable analysis (Table 2). All variables significant in univariable analysis (*p* < 0.05) were entered simultaneously into the multivariable model. In multivariable analysis (Table 3; Fig. 2), older age (OR 1.048, 95% CI 1.037–1.059, *p* < 0.001) was strongly associated with increased odds of IGRA positivity. Among comorbidities, pulmonary nodules remained independently associated (OR 1.608, 95% CI 1.094–2.365, *p* = 0.016). Among laboratory parameters, higher eosinophil percentage was independently associated with increased odds (OR 1.109, 95% CI 1.033–1.190, *p* = 0.004), whereas platelet count was not significant after adjustment (OR 0.999, 95% CI 0.998–1.001, *p* = 0.574). Regarding active tuberculosis, in the available medical records of the included analytic cohort, no microbiologically confirmed active tuberculosis was documented. Microbiological testing for active tuberculosis was performed when clinically indicated rather than systematically in all participants. Biologic exposure was associated with reduced odds of IGRA positivity (OR 0.705, 95% CI 0.512–0.969, *p* = 0.031). To further explore the potential explanation for this association, we compared baseline characteristics between patients with and without prior biologic exposure. Patients with prior biologic exposure were younger than those without prior biologic exposure (mean age, 40.0 ± 15.6 years vs. 43.8 ± 16.3 years, respectively; *p* < 0.001), whereas the prevalence of pulmonary nodules was similar between the two groups [126/828 (15.2%) vs. 43/298 (14.4%), respectively; *p* = 0.817] (Supplementary Table 1). Other variables significant in univariable analysis, including hypertension, diabetes mellitus, and disease duration ≥ 10 years, were no longer significant after adjustment, suggesting that their associations with IGRA positivity may have been influenced by other covariates.

**Table 2:** Univariable logistic regression: factors associated with IGRA positivity (*n* = 1126)

Variable	Crude OR	95% CI	*p* value
***Demographics***			
Age (per year)	1.050	1.040–1.060	**< 0.001**
Male sex	1.348	0.997–1.823	0.052
BMI (per kg/m²)	1.000	0.969–1.032	0.995
***Psoriasis characteristics***			
Disease duration ≥ 10 years	1.490	1.107–2.006	**0.009**
***Comorbidities***			
Hypertension	2.003	1.363–2.944	**< 0.001**
Diabetes mellitus	2.237	1.392–3.597	**< 0.001**
Pulmonary nodules	2.296	1.630–3.234	**< 0.001**
***Laboratory parameters***			
Eosinophil (per %)	1.130	1.056–1.209	**< 0.001**
Platelet (per ×10⁹/L)	0.998	0.996–1.000	**0.025**
NLR	1.058	0.958–1.169	0.265
PLR	1.000	0.998–1.002	0.995
MLR	3.740	1.155–12.109	**0.028**
ELR	22.557	5.036–101.033	**< 0.001**
***Treatment***			
Prior biologic exposure	0.609	0.455–0.815	**< 0.001**

**Table 3:** Multivariable logistic regression analysis of factors associated with IGRA positivity (*n* = 1126)

Variable	Adjusted OR	95% CI	*p* value
***Demographics***			
Age (per year)	1.048	1.037–1.059	**< 0.001**
Male sexᵃ	—	—	—
BMI (per kg/m²)ᵃ	—	—	—
***Psoriasis characteristics***			
Disease duration ≥ 10 years	1.147	0.829–1.587	0.409
***Comorbidities***			
Hypertension	0.755	0.475–1.202	0.237
Diabetes mellitus	1.062	0.616–1.830	0.829
Pulmonary nodules	1.608	1.094–2.365	**0.016**
***Laboratory parameters***			
Eosinophil (per %)	1.109	1.033–1.190	**0.004**
Platelet (per ×10⁹/L)	0.999	0.998–1.001	0.574
NLR, PLR, MLR, ELRᵃ ᵇ	—	—	—
***Treatment***			
Prior biologic exposure	0.705	0.512–0.969	**0.031**

Variables with *p* < 0.05 in univariable analysis were entered into the multivariable model. ᵃ Male sex, BMI, NLR, and PLR were not included (univariable *p* ≥ 0.05). ᵇ MLR and ELR were excluded because of collinearity with eosinophil percentage. Bold indicates *p* < 0.05

### IGRA positivity across psoriasis subtypes

**Fig. 3:**
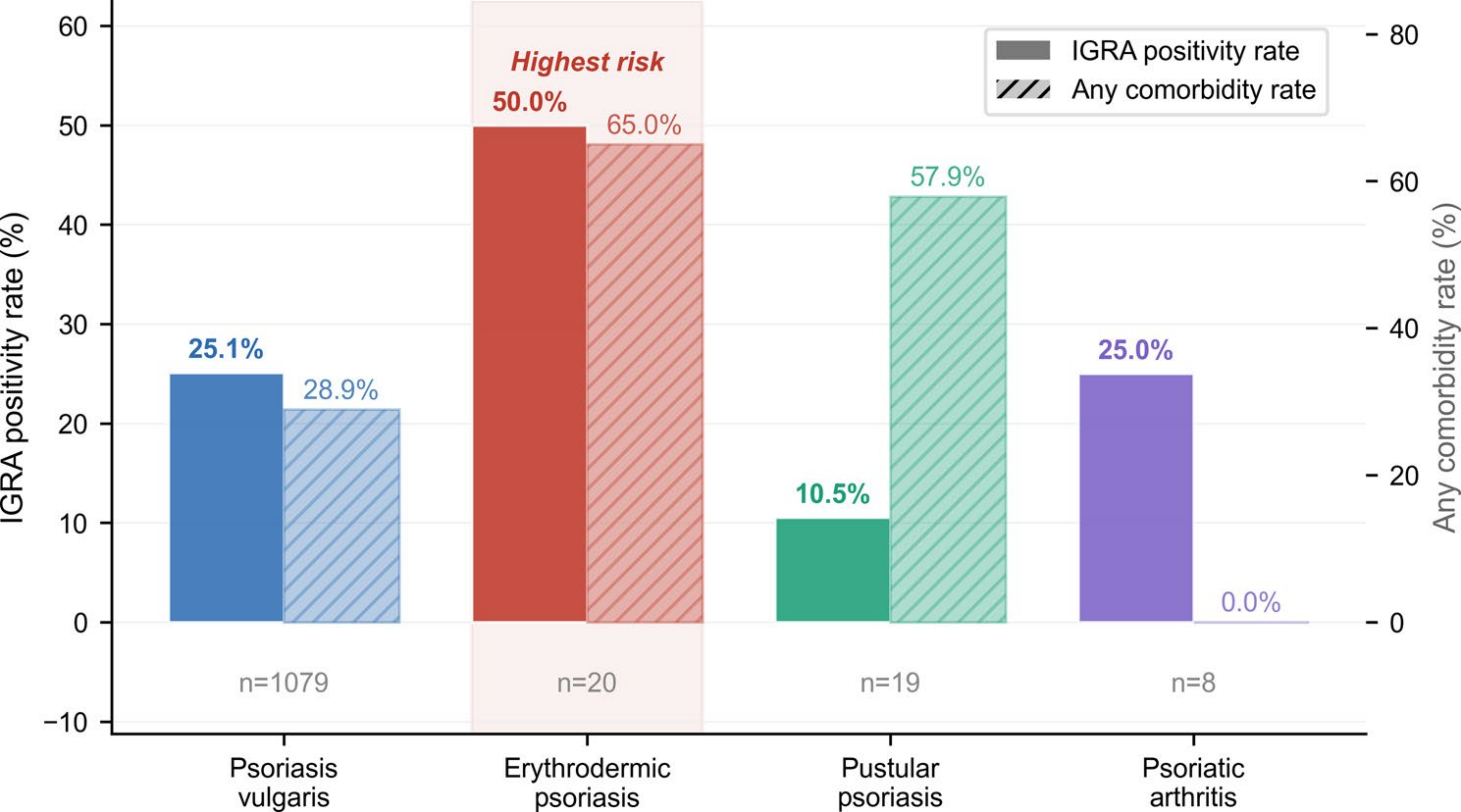
IGRA positivity and any comorbidity rates by psoriasis subtype. Comparison of IGRA positivity rates and any comorbidity rates among patients with psoriasis vulgaris, erythrodermic psoriasis, psoriatic arthritis, and pustular psoriasis. “Any comorbidity” was defined as the presence of at least one variable listed under the Comorbidities category in Table 1, including hypertension, diabetes mellitus, fatty liver disease, hyperlipidemia, pulmonary nodules, or liver dysfunction

IGRA positivity differed significantly across psoriasis subtypes (*p* = 0.034; Table 4; Fig. 3). Erythrodermic psoriasis showed the highest positivity rate (50.0%), followed by psoriasis vulgaris (25.1%), psoriatic arthritis (25.0%), and pustular psoriasis (10.5%). As shown in Fig. 3, the markedly higher IGRA positivity observed in erythrodermic psoriasis contrasted with the substantially lower rate in pustular psoriasis, whereas psoriasis vulgaris and psoriatic arthritis showed comparable intermediate levels. Detailed clinical characteristics across subtypes are presented in Table 4. Notably, erythrodermic psoriasis was associated with an older age distribution, longer disease duration, and a higher prevalence of comorbidities and pulmonary nodules compared with other subtypes. 

**Table 4:** Clinical characteristics and IGRA positivity across psoriasis subtypes (*n* = 1126)

Variable	Psoriasis vulgaris (*n* = 1079)	Erythrodermic psoriasis (*n* = 20)	Pustular psoriasis (*n* = 19)	Psoriatic arthritis (*n* = 8)	*p* value
IGRA positive, n (%)	271 (25.1)	10 (50.0)	2 (10.5)	2 (25.0)	**0.034**
***Demographics***					
Age, years	40.6 ± 15.9	51.5 ± 12.4	46.7 ± 17.1	47.0 ± 10.3	**0.003**
Male sex, n (%)	754 (69.9)	13 (65.0)	9 (47.4)	6 (75.0)	0.190
BMI (kg/m²)	26.2 ± 4.3	22.2 ± 3.4	25.3 ± 3.6	25.7 ± 3.7	**< 0.001**
***Psoriasis characteristics***					
Disease duration, years	11.8 ± 8.5	18.6 ± 11.9	13.2 ± 11.7	15.2 ± 11.6	**0.019**
Duration ≥ 10 years, n (%)	715 (66.3)	17 (85.0)	12 (63.2)	6 (75.0)	0.327
Prior biologic exposure, n (%)	802 (74.3)	12 (60.0)	8 (42.1)	6 (75.0)	**0.008**
***Comorbidities***					
Any comorbidity, n (%)	312 (28.9)	13 (65.0)	11 (57.9)	0 (0.0)	**< 0.001**
Hypertension, n (%)	118 (10.9)	6 (30.0)	4 (21.1)	0 (0.0)	**0.020**
Diabetes mellitus, n (%)	70 (6.5)	4 (20.0)	3 (15.8)	0 (0.0)	**0.035**
Pulmonary nodules, n (%)	156 (14.5)	8 (40.0)	5 (26.3)	0 (0.0)	**0.004**
***Laboratory parameters***					
Platelet (×10⁹/L)	259.7 ± 72.1	339.4 ± 91.3	276.9 ± 112.1	257.6 ± 87.4	**0.001**
Lymphocyte (%)	29.7 ± 8.5	20.5 ± 7.8	28.9 ± 8.4	25.4 ± 6.8	**< 0.001**
Eosinophil (%)	2.5 ± 1.9	2.8 ± 2.6	2.1 ± 1.5	2.7 ± 1.6	0.570
WBC (×10⁹/L)	7.1 ± 2.1	7.6 ± 1.6	7.3 ± 2.6	7.6 ± 3.0	0.325

Data presented as mean ± SD or n (%). p values: Kruskal–Wallis test (continuous); chi-square test (categorical). Bold p values: *p* < 0.05

### Model performance and decision curve analysis

**Fig. 4:**
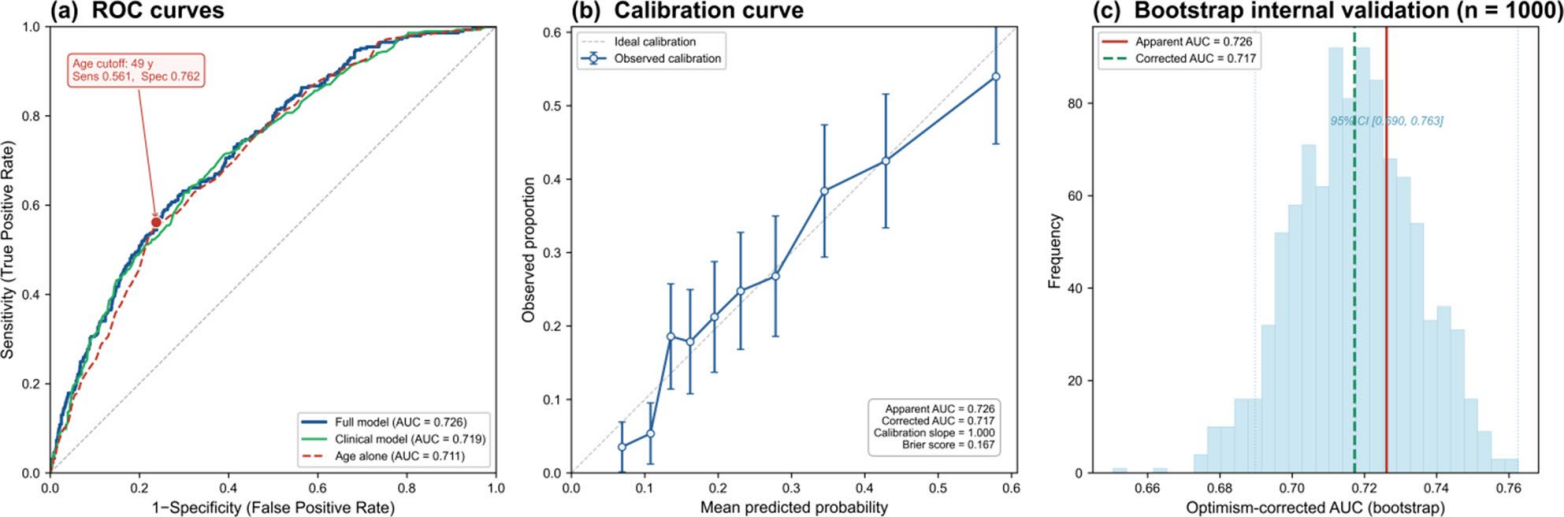
ROC analysis, model calibration, and bootstrap internal validation. (**a**) Receiver operating characteristic (ROC) curves for the eight-variable full model, the corresponding six-variable clinical model, and age alone. (**b**) Calibration curve showing agreement between predicted and observed probabilities. (**c**) Bootstrap validation results. AUC, area under the curve; CI, confidence interval

The eight-variable multivariable model demonstrated acceptable discrimination, with an AUC of 0.726 (Fig. 4a), and good calibration (Brier score 0.167). The model included age, disease duration ≥ 10 years, hypertension, diabetes mellitus, pulmonary nodules, prior biologic exposure, platelet count, and eosinophil percentage; the full model specification is provided in the Supplementary Material. The calibration curve showed good agreement between predicted and observed IGRA positivity (Fig. 4b). After internal validation using 1,000 bootstrap resamples, the model showed an optimism of 0.009, yielding an optimism-corrected C-statistic of 0.717 (95% CI 0.690–0.763) (Fig. 4c), indicating stable internal performance with limited overfitting. Among the individual predictors, age showed the highest discriminatory ability, with an AUC of 0.711. 

**Fig. 5:**
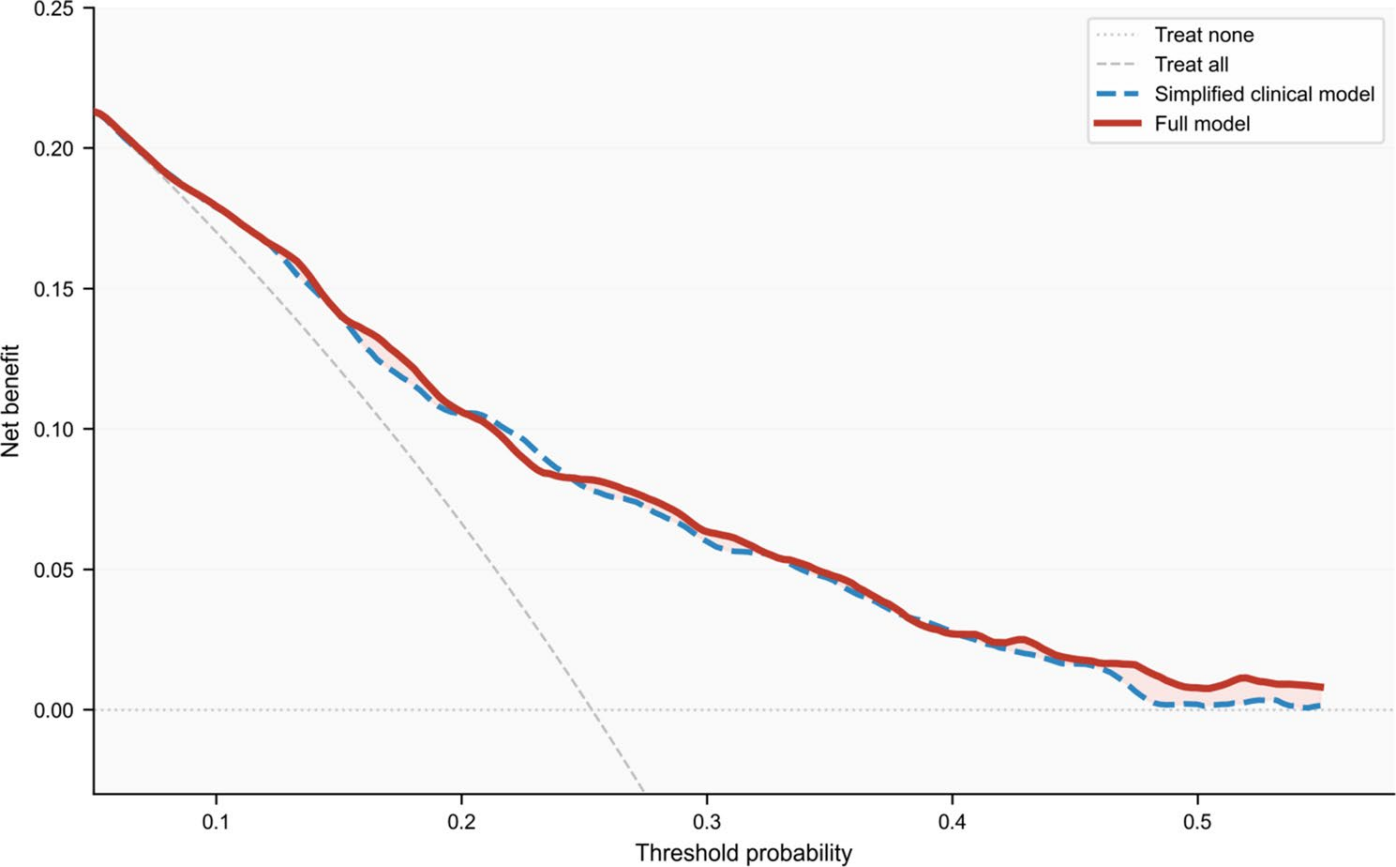
Clinical utility of the prediction model assessed by decision curve analysis. Net clinical benefit across a range of threshold probabilities. The full model included age, disease duration ≥10 years, hypertension, diabetes mellitus, pulmonary nodules, prior biologic exposure, platelet count, and eosinophil percentage. The clinical model excluded the two laboratory variables and included the remaining six non-laboratory variables

Decision curve analysis showed that both the clinical model and the full model provided greater net benefit than the “treat all” and “treat none” strategies across the clinically relevant threshold range of approximately 5%–50% (Fig. 5). The full model showed slightly greater net benefit than the clinical model at most thresholds, suggesting modest additional clinical utility.

## Discussion

Among 1,126 patients with psoriasis without documented prior tuberculosis, older age, pulmonary nodules, and a higher eosinophil percentage were independently associated with IGRA positivity, whereas prior biologic exposure showed an inverse association. The model demonstrated moderate discrimination and reasonable calibration after internal validation. Older age and pulmonary nodules are clinically plausible findings rather than unexpected discoveries; the contribution of this study lies in quantifying these associations in a large real-world psoriasis cohort and integrating routinely available characteristics into an internally validated framework for estimating the likelihood of IGRA positivity. Psoriasis is increasingly recognized as a systemic inflammatory disease characterized by complex interactions between immune dysregulation and comorbidities, which may contribute to heterogeneity in clinical and laboratory findings [19].

Older age was the strongest continuous predictor of IGRA positivity in our cohort. The positivity rate increased substantially with age, from 8.2% in patients aged ≤ 30 years to 45.6% in those aged > 60 years. Age also showed the highest individual discriminative ability among the candidate predictors, and 49 years was identified as the optimal cutoff value. This finding is in line with recent evidence showing that LTBI/IGRA positivity becomes more common with advancing age, likely reflecting cumulative lifetime exposure to Mycobacterium tuberculosis; in addition, more advanced age has remained an independent correlate of prevalent LTBI in contemporary Asian cohorts [20]. Clinically, these findings suggest that age should be considered an important component of TB risk stratification before biologic initiation in patients with psoriasis.

Patients with a documented history of prior tuberculosis were deliberately excluded from the analytic cohort because IGRA results in this subgroup predominantly reflect known previous infection rather than the screening scenario in which the model is intended to be applied. By restricting the development cohort to patients without a history of prior tuberculosis, the model was tailored to estimate the probability of IGRA positivity among patients undergoing tuberculosis assessment in routine dermatological care [21]. Nonetheless, a thorough history of prior tuberculosis remains an essential component of pre-biologic evaluation, as patients with documented prior TB are routinely managed as high-risk regardless of any prediction model. In practice, underreporting should also be considered, particularly in older patients or in those with incomplete historical medical records.

Pulmonary nodules were also independently associated with IGRA positivity. One possible explanation is that prior mycobacterial exposure may leave residual radiologic findings that coexist with persistent immune sensitization. Recent screening studies in other biologic-treated inflammatory diseases suggest that chest imaging remains part of many pre-biologic LTBI algorithms, although its incremental value over immunological assays may depend strongly on the background TB burden of the setting [22, 23]. In our study, pulmonary nodules were more common in IGRA-positive patients than in IGRA-negative patients. This finding supports the potential value of incorporating chest imaging findings into pre-treatment TB risk assessment, especially in regions with an intermediate or high TB burden. However, the extent to which additional imaging-based evaluation improves screening performance requires further investigation.

Another notable finding was the independent association between eosinophil percentage and IGRA positivity. To our knowledge, this variable has rarely been reported in psoriasis populations undergoing TB screening prior to biologic therapy. Several explanations may be considered. Rather than implying a direct causal role, a higher eosinophil percentage may reflect broader immune dysregulation in psoriasis. In addition, experimental and translational studies in tuberculosis have demonstrated that eosinophils can participate in host immune responses to Mycobacterium tuberculosis, indicating a potential, although indirect, link to IGRA reactivity. Alternatively, eosinophil elevation may serve as a nonspecific marker of systemic inflammatory activity rather than a direct mechanistic contributor. The higher ELR observed in the unadjusted analyses was directionally consistent with this finding; however, ELR was excluded from the multivariable model because of collinearity with eosinophil percentage. Nevertheless, the biological basis underlying this association remains uncertain and warrants further investigation.

Prior use of biologic agents was associated with lower odds of IGRA positivity in the present study. This finding should be interpreted cautiously and should not be construed as indicating a protective effect of biologic therapy against tuberculosis infection. A more plausible explanation is that the association reflects clinical management pathways and patient selection. In clinical practice, patients with positive IGRA results generally undergo further evaluation to exclude active tuberculosis and, when appropriate, receive preventive treatment before biologic therapy is initiated. Such patients may therefore experience delayed treatment, may be preferentially prescribed biologic classes with a lower risk of tuberculosis reactivation, or may be less likely to have received biologic therapy before the IGRA assessment captured in this study. Current evidence also suggests that tuberculosis reactivation risk varies across biologic classes: tumor necrosis factor inhibitors remain the primary concern, whereas interleukin-17 and interleukin-23 inhibitors appear to carry substantially lower risk, particularly in patients without additional risk factors [24]. Furthermore, recent real-world data and expert recommendations have questioned the value of indiscriminate repeat tuberculosis testing in all patients receiving biologic therapies, especially in low-risk settings [25]. Given the cross-sectional design, however, the temporal relationship between IGRA testing and biologic treatment could not be determined, and baseline IGRA results at biologic initiation were unavailable for some patients. In addition, IGRA positivity has limited predictive value for progression to active tuberculosis and may fluctuate over time, particularly near the assay threshold [26]. Taken together, the observed inverse association is more likely to reflect clinical decision-making and selection patterns than a true biological effect of biologic agents. Consistent with this interpretation, patients with prior biologic exposure were younger than those without prior biologic exposure, suggesting that baseline differences and treatment selection may have contributed to the association.

We also observed significant differences in IGRA positivity across psoriasis subtypes. Erythrodermic psoriasis showed the highest positivity rate, whereas pustular psoriasis showed the lowest. These differences may largely reflect baseline variation in age, comorbidity burden, pulmonary nodules, and treatment exposure across subtypes. Erythrodermic psoriasis is recognized as an uncommon and severe systemic inflammatory phenotype, as described in recent reviews [27, 28]. However, because the non-vulgaris subtype groups were small and subtype was not evaluated as an independent factor in the multivariable model, these findings should be regarded as exploratory and should not be interpreted as evidence of an independent subtype effect.

The overall performance of the multivariable model was acceptable but moderate. The apparent AUC was 0.726, and the optimism-corrected AUC after bootstrap validation was 0.717, suggesting relatively stable internal performance with limited overfitting. Calibration analysis also showed reasonable agreement between predicted and observed IGRA positivity. Decision curve analysis suggested potential net benefit across the modeled threshold range of approximately 5% to 50%; however, this finding should be considered exploratory because the model was not designed to determine whether IGRA testing or chest imaging should be performed. Instead, the model may provide supplementary context for patients undergoing tuberculosis assessment in relation to biologic therapy, particularly when clinical characteristics and IGRA results appear discordant. A low predicted probability should not override a positive IGRA result, whereas a high predicted probability alone should not be regarded as evidence of tuberculosis infection. External validation and prospective impact studies are required before the model can be used to guide clinical decisions [29].

This study has several limitations. First, it was a single-center retrospective study, which may limit the generalizability of the findings. Second, the cross-sectional design precludes causal inference, and the model should therefore be interpreted as a framework for estimating the probability of IGRA positivity rather than for etiologic inference or prediction of future tuberculosis. Third, IGRA positivity reflects immune sensitization to Mycobacterium tuberculosis but cannot reliably distinguish latent infection from active tuberculosis or previous infection from current infection. Baseline IGRA results at the initiation of previous biologic therapy were unavailable for some patients because of the retrospective design, which limited assessment of the temporal relationship between biologic exposure and subsequent IGRA positivity. In addition, microbiological testing for active tuberculosis was performed only when clinically indicated rather than systematically in all participants, and post-referral diagnostic information from specialized tuberculosis centers was not uniformly available. Therefore, we cannot exclude the possibility that a small number of patients with active tuberculosis were not identified in the available records. Current tests for tuberculosis infection also have limited ability to identify which individuals will progress to active disease, and serial-testing studies have shown that IGRA results may fluctuate over time, particularly when values are close to the assay cutoff [30]. Fourth, patients with a documented history of prior tuberculosis (*n* = 10) were excluded from the primary analysis. Although this design choice was intended to align the development cohort with the clinical setting in which probability estimation may be most relevant, it also means that the model is not applicable to patients with known prior tuberculosis and should be interpreted alongside a thorough tuberculosis history. Fifth, some psoriasis subtypes were represented by relatively small sample sizes, reducing the robustness of subgroup comparisons. Finally, this was an internally validated, single-center model; external validation in independent cohorts with different clinical settings and tuberculosis epidemiological backgrounds, followed by prospective impact assessment, is required before the model can be considered for clinical decision-making [31]. In conclusion, older age, pulmonary nodules, and a higher eosinophil percentage were associated with IGRA positivity in patients with psoriasis without documented prior tuberculosis, whereas prior biologic exposure showed an inverse association. The model showed moderate internal performance and may provide supplementary clinical context, but it should not replace standard tuberculosis assessment.

## Supplementary Information

Below is the link to the electronic supplementary material.

Supplementary Material 1

## Data Availability

The datasets generated and/or analysed during the current study are not publicly available due to privacy and ethical restrictions, but are available from the corresponding author on reasonable request and with permission from the relevant institutional authority.
