# Supplementary material for: Factors associated with interferon-gamma release assay positivity in patients with psoriasis: a cross-sectional study
Source: BMC Infect Dis. 2026 Jul 29;26:1495. doi: 10.1186/s12879-026-14090-z (PMC13474803; doi:10.1186/s12879-026-14090-z)
Supplement: Supplementary file 1 [file 12879_2026_14090_MOESM1_ESM.docx]

## **Supplementary Material**

**1. Full specification of the final multivariable logistic regression model**

## The primary full model included eight variables: age, disease duration ≥10 years, hypertension, diabetes mellitus, pulmonary nodules, prior biologic exposure, platelet count, and eosinophil percentage. The model was specified as follows:

## **logit(*P*) = −3.137249 + 0.046425 × Age + 0.136871 × Disease duration ≥10 years − 0.280398 × Hypertension + 0.060023 × Diabetes mellitus + 0.475142 × Pulmonary nodules − 0.350192 × Prior biologic exposure − 0.000556 × Platelet count + 0.103500 × Eosinophil percentage.**

## Here, *P* represents the predicted probability of IGRA positivity. Age was entered in years, platelet count in ×10⁹/L, and eosinophil percentage as a continuous variable. Disease duration ≥10 years, hypertension, diabetes mellitus, pulmonary nodules, and prior biologic exposure were coded as binary indicators (1 = yes; 0 = no). The predicted probability of IGRA positivity can be calculated as follows:

## ***P* = exp[logit(P)]/{1 + exp[logit(P)]}.**

## The simplified clinical model was used as a comparator in the receiver operating characteristic and decision curve analyses. It included the six non-laboratory variables: age, disease duration ≥10 years, hypertension, diabetes mellitus, pulmonary nodules, and prior biologic exposure.

## **2.Supplementary methods: missing data handling**

## Missing data were handled using multiple imputation by chained equations under the assumption of missing at random. All candidate predictors and the outcome were included in the imputation model to minimize potential bias. Multiple imputed datasets were generated, and results were pooled across imputed datasets according to Rubin’s rules.

**3.Supplementary Table 1. Comparison of baseline characteristics according to prior biologic exposure**

| **Variable** | **Prior biologic exposure (n = 828)** | **No prior biologic exposure (n = 298)** | ***p* value** |
| --- | --- | --- | --- |
| Age, years | 40.0 ± 15.6 | 43.8 ± 16.3 | 0.001 |
| Pulmonary nodules | 126 (15.2) | 43 (14.4) | 0.817 |

Data are presented as mean ± standard deviation or n (%). The *p* values were calculated using the independent-samples *t* test for age and the chi-square test for pulmonary nodules.

**4.Supplementary Table 2. Association between absolute eosinophil count and IGRA positivity**

| **Variable** | **IGRA-negative (n = 841)** | **IGRA-positive (n = 285)** | ***p* value** |
| --- | --- | --- | --- |
| Absolute eosinophil count, ×10⁹/L | 0.13 (0.08-0.22) | 0.15 (0.09-0.26) | 0.009 |

| **Variable** | **Crude OR** | **95% CI** | ***p* value** | **Adjusted OR** | **95% CI** | ***p* value** |
| --- | --- | --- | --- | --- | --- | --- |
| Absolute eosinophil count, per 0.1 ×10⁹/L increase | 1.170 | 1.070-1.280 | 0.001 | 1.173 | 1.065-1.293 | 0.001 |

**Note:** Data are presented as median (IQR). The *p* value for group comparison was calculated using the *Mann-Whitney U* test. In the multivariable logistic regression model, eosinophil percentage was replaced by absolute eosinophil count, and the model was adjusted for age, disease duration ≥10 years, hypertension, diabetes mellitus, pulmonary nodules, platelet count, and prior biologic exposure. OR, odds ratio; CI, confidence interval; IGRA, interferon-gamma release assay.
